# Therapeutic Potential and Immunomodulatory Role of Coenzyme Q_10_ and Its Analogues in Systemic Autoimmune Diseases

**DOI:** 10.3390/antiox10040600

**Published:** 2021-04-13

**Authors:** Chary López-Pedrera, José Manuel Villalba, Alejandra Mª Patiño-Trives, Maria Luque-Tévar, Nuria Barbarroja, Mª Ángeles Aguirre, Alejandro Escudero-Contreras, Carlos Pérez-Sánchez

**Affiliations:** 1 Rheumatology Service, Reina Sofia Hospital/Maimonides Institute for Research in Biomedicine of Córdoba (IMIBIC), University of Córdoba, 14004 Córdoba, Spain; alejandra.patino@imibic.org (A.M.P.-T.); maria.luque@imibic.org (M.L.-T.); nuria.barbarroja.exts@juntadeandalucia.es (N.B.); angeles.aguirre.sspa@juntadeandalucia.es (M.Á.A.); alejandro.escudero.sspa@juntadeandalucia.es (A.E.-C.); 2 Department of Cell Biology, Immunology and Physiology, Agrifood Campus of International Excellence, University of Córdoba, ceiA3, 14014 Córdoba, Spain; jmvillalba@uco.es (J.M.V.); b32pesac@uco.es (C.P.-S.)

**Keywords:** coenzyme Q10, autoimmune disorders, inflammation, cardiovascular disease

## Abstract

Coenzyme Q_10_ (CoQ_10_) is a mitochondrial electron carrier and a powerful lipophilic antioxidant located in membranes and plasma lipoproteins. CoQ_10_ is endogenously synthesized and obtained from the diet, which has raised interest in its therapeutic potential against pathologies related to mitochondrial dysfunction and enhanced oxidative stress. Novel formulations of solubilized CoQ_10_ and the stabilization of reduced CoQ_10_ (ubiquinol) have improved its bioavailability and efficacy. Synthetic analogues with increased solubility, such as idebenone, or accumulated selectively in mitochondria, such as MitoQ, have also demonstrated promising properties. CoQ_10_ has shown beneficial effects in autoimmune diseases. Leukocytes from antiphospholipid syndrome (APS) patients exhibit an oxidative perturbation closely related to the prothrombotic status. In vivo ubiquinol supplementation in APS modulated the overexpression of inflammatory and thrombotic risk-markers. Mitochondrial abnormalities also contribute to immune dysregulation and organ damage in systemic lupus erythematosus (SLE). Idebenone and MitoQ improved clinical and immunological features of lupus-like disease in mice. Clinical trials and experimental models have further demonstrated a therapeutic role for CoQ_10_ in Rheumatoid Arthritis, multiple sclerosis and type 1 diabetes. This review summarizes the effects of CoQ_10_ and its analogs in modulating processes involved in autoimmune disorders, highlighting the potential of these therapeutic approaches for patients with immune-mediated diseases.

## 1. Introduction to Coenzyme Q and Coenzyme Q-Related Compounds

### 1.1. Basic Principles

Coenzyme Q (CoQ) is a prenylated para-benzoquinone that is found ubiquitously in aerobic organisms and is also present in all cells and tissues and in plasma lipoproteins, hence its denomination as ubiquinone [1,2]. CoQ isoforms are designated according to the number of isoprene units in the side chain with CoQ_10_ being the main isoform present in humans [3]. Those organs with high-energy requirements or metabolic activity such as the heart, kidney, liver and skeletal muscle contain the highest levels of CoQ [4]. The redox activity of CoQ resides in the quinone groups that can be reduced to the corresponding hydroquinone to yield reduced CoQ, also known as ubiquinol, which can be oxidized to regenerate ubiquinone [5]. The majority of CoQ in tissues and plasma is found as ubiquinol, except for some organs such as brain and lungs, which appears to be related to increased oxidative stress in these two tissues [4].

In the inner mitochondrial membrane CoQ mediates electron transport coupled to ATP synthesis [6,7]. Mitochondrial CoQ also participates in fatty acid β-oxidation, amino acid catabolism, pyrimidine biosynthesis, modulation of mitochondrial uncoupling proteins (UCPs) sensitivity to purine nucleotides, and the regulation of the activity of the mitochondrial permeability transition pore [7]. At the plasma membrane, CoQ participates in a trans-plasma membrane redox system that has been related to the control of cell growth, the reoxidation of cellular nicotinamide adenine dinucleotide (NADH) under conditions of mitochondrial dysfunction, and the regeneration of ascorbate in the extracellular space [8,9]. Another prominent function of CoQ (in its reduced form) is to provide antioxidant protection within the lipid phase of membranes and plasma lipoproteins, either directly or through the regeneration of α-tocopherol [3,7,8,9]. CoQ is also a potent stabilizer of membranes [10].

Whereas for some of these functions CoQ behaves as a vitamin-like molecule and its structure resembles that of vitamin K, it cannot be classified as such since it can be synthesized endogenously [4]. In fact, CoQ is the only lipid-soluble antioxidant that can be synthesized by all aerobic organisms, from bacteria to humans [2,3]. Highest rates of CoQ biosynthesis are observed in metabolically active tissues such as the heart and immune system [11]. Mutations in several human CoQ_10_ biosynthesis genes result in primary CoQ_10_ deficiencies, but other genetic defects can also produce secondary CoQ_10_-deficiencies [12,13,14]. Endogenous biosynthesis can also be impaired under several pathological situations, and a deficiency in CoQ_10_ is also involved in cardiomyopathies and degenerative muscle and neuronal diseases [15,16,17,18]. The synthesis of CoQ_10_ may also be compromised by aging [19] and by the action of some pharmacological agents such as statins [20].

### 1.2. Pharmacokinetics of Coenzyme Q_10_

CoQ_10_ is present in a normal diet, but its amount in dairy products is very low and it is poorly absorbed [11,21]. In the absence of an effective supplementation strategy, dietary CoQ_10_ is not sufficient to support its physiological functions and does not appear to contribute to the regulation of CoQ_10_ levels in tissues and in plasma, which, under normal circumstances, are determined by the rate of its endogenous biosynthesis taking place in all tissues [22]. Augmentation of CoQ_10_ levels by dietary supplementation may ameliorate the deleterious consequences of CoQ_10_ deficiencies, and CoQ_10_ is also currently a popular over-the-counter dietary supplement. However, orally administered CoQ_10_ is also poorly absorbed. Although the absorption of CoQ_10_ can be enhanced by the presence of lipids in the diet, only a minor proportion of the CoQ_10_ that is ingested is effectively absorbed [4]. In consequence, the percentage of supplemental CoQ_10_ that is effectively incorporated decreases as the supplement increases, and those doses that exceed that capacity for absorption have a minimal effect on efficacy [23]. While the use of high doses of CoQ_10_ appears thus unnecessary because it increases the cost of the treatment without significant benefits, there is an excellent safety record for CoQ_10_ [24,25,26,27]. Moreover, exogenous CoQ_10_ does not affect its endogenous biosynthesis, nor does it accumulate into plasma or tissues once supplementation has ceased [15].

### 1.3. Coenzyme Q_10_ Formulations with Enhanced Bioavailability and Coenzyme Q10-Related Compounds with Therapeutic Interest in Autoimmune Diseases

The poor bioavailability of CoQ_10_ has prompted the search of formulations that improve its absorption and delivery to the target compartments. The uptake of CoQ_10_ is further compromised by its instability to light and thermolability [11,28]. Efforts to enhance CoQ_10_ bioavailability have mainly been directed towards the reduction in particle size and the increase of water solubility/dispersibility using complexation, solubilization, or reduction [29]. Very promising results using modified-release preparations have also been reported [30]. The different procedures to increase the bioavailability and stability of CoQ_10_ in formulations have been revised recently [11,31], and include liposomes, lipid-free nano-formulation, water-soluble CoQ_10_ complexes with β-cyclodextrin, self-nanoemulsifying CoQ_10_, CoQ_10_-loaded oleo-gels, and CoQ_10_ micellization with caspofungin [31]. In general, the relative bioavailability of CoQ_10_ is strongly dependent on the delivery system, with the order of decreasing bioavailability being: nanoparticulated, solubilized, oil-emulsioned and powder [12]. Absorption of CoQ_10_ is also substantially improved when provided in the form of ubiquinol [25,26,27,28,29,30,31,32]

Another attempt to overcome the low bioavailability of CoQ_10_ has been represented by the development of short-chain analogues that can be easily delivered to those sites where improvement of CoQ_10_ function is needed. These analogues include molecules such as MitoQ and the SkQ series that accumulate specifically in the mitochondria driven by the electrochemical potential, as well as molecules such as idebenone and EPI-743 which are not targeted to the mitochondria but shuttle between this organelle and the cytosol for the regeneration of their active hydro-quinones [12,33]. These analogues have already been tested in clinical assays with promising results for the treatment of several diseases associated with the impairment of mitochondrial function and, among them, idebenone and MitoQ have been tested in the treatment of autoimmune diseases (see below).

## 2. Relevance of Oxidative Stress and Mitochondrial Dysfunction in the Physiopathology of Autoimmune Disorders

### 2.1. Oxidative Stress, Mitochondrial Dysfunction and Disease

Molecular oxygen is the driving force behind oxidative phosphorylation. Through this process, mitochondria use multi-subunit enzyme complexes to oxidize nutrients and use the released energy to form adenosine triphosphate (ATP). This absolutely vital function is, however, inevitably connected to the creation of highly reactive and toxic side products, the reactive oxygen species (ROS) [34].

It has been demonstrated that ROS are not only by-products but can also act as secondary messengers. Thus, mitochondrial ROS are not simply the price we pay for respiration, but also essential for regulating mitochondrial function and physiology. Besides, ROS are not only produced during oxidative phosphorylation, but also, and in even higher amounts, by NADPH oxidase complexes (NOX). The best described and most potent of these complexes is NOX2, which is predominantly expressed in phagocytic cells such as neutrophils, monocytes and macrophages, or dendritic cells (DC), and is the dominant ROS-generating complex in mammals [35].

Both reactive oxygen and nitrogen species (RNS), mainly represented by nitric oxide (^∂^NO), superoxide anion radical (^∂^O_2_^−^), peroxy-nitrite anion (ONOO^−^), hydroxyl (^∂^OH), lipo-peroxide (LOO^∂^), hydrogen peroxide (H_2_O_2_), and hypochlorous acid (HClO), are highly reactive molecules generated both during physiological cellular processes and under several pathological conditions [36,37,38,39]. In addition to the presence of increased amounts of ROS/RNS in conditions of oxidative stress, antioxidants can behave as regulatory players, as they are capable of scavenging ROS/RNS, thus inhibiting the cell oxidation [40]. Antioxidants encompass enzymatic and nonenzymatic systems. The enzyme system primarily includes catalase (CAT), superoxide dismutase (SOD), glutathione peroxidase (GPx), glutathione reductase (GR) and thioredoxin reductase. The nonenzymatic antioxidant system contains some vitamins [41].

The main function of mitochondria is the production of ATP through oxidative phosphorylation. In addition, it is involved in the generation and detoxification of ROS, apoptosis, synthesis and catabolism of metabolites, regulation of mitochondrial and cytoplasmic matrix calcium, and transport of organelles themselves to the correct location inside the cells. Any anomaly in these processes can be named mitochondrial dysfunction [42].

There are different ways to assess mitochondrial dysfunction depending on whether function is determined in isolated mitochondria or in intact cells. The most simple and useful test to identify energetic dysfunction in isolated mitochondria is to measure mitochondrial respiratory control. The measurement of the protonmotive force (pmf) and the identification of respiration rate under different conditions and states can clarify the mechanism. However, the best way to obtain mechanical insights is to titrate both respiration rate and pmf at the same time, and draw dynamic diagrams of differential oxidative phosphorylation [43].

In intact cells, the analysis of cell respiratory control is the most useful test to evaluate mitochondrial function. That testing yields information that allows a quick, simple and full assessment of the bioenergetic status of the cells: how fast they are turning over ATP, how well-coupled their mitochondria are, and how much spare respiratory capacity they have available to deal with energetic demands. The determination of the mitochondrial membrane potential (ΔΨm) can also be very helpful. Mitochondrial membrane potential is a key marker of mitochondrial activity, as it reveals the electron transport process and oxidative phosphorylation, which is the energetic force for ATP production. Thus, the ΔΨm is a major indicator of cell health or damage [44].

Ultimately, mitochondrial dysfunction can be assessed by analyzing its ‘dynamics’. Mitochondria experience coordinated cycles of fission and fusion, commonly known as ‘mitochondrial dynamics’ to preserve their shape, size and distribution. Mitochondrial fusion leads to tubular, elongated, and interconnected mitochondrial nets. This process is under the control of the fusion proteins mitofusin (Mfn-1, Mfn-2), and optic atrophy protein 1. On the contrary, mitochondrial fission generates divided and discontinuous mitochondria in a process coordinated by the mitochondrial fission proteins dynamin-related protein 1 (Drp1) and the human fission protein 1 (hFis1) [45].

Functionally, mitochondrial fission and fusion may modify the consumption of oxygen, mitochondrial respiration and the mitochondrial membrane potential. Besides, mitochondrial dynamics are crucial for a variety of biological processes such as apoptosis, respiration, mitosis, and development. Moreover, numerous studies have reported abnormal mitochondrial morphology, impacting on a broad range of pathological processes and associated with a number of human diseases [46]. Finally, mitochondrial biogenesis, the complex cellular process of new mitochondria generation, has a key role in boosting mitochondrial function. Together with fission/fusion, biogenesis is vital for the homeostasis of the mitochondria, for the exclusion and elimination of damaged mitochondria and, fundamentally, for the regeneration of the mitochondrial network in the cells [47].

**Figure 1:**
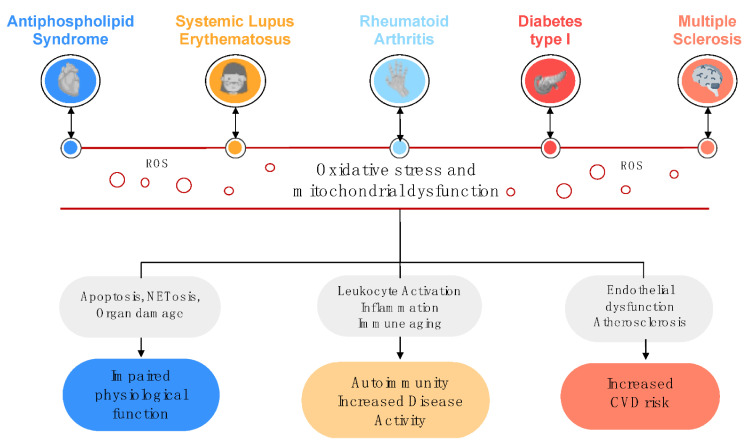
Pathogenic role of oxidative stress and mitochondrial dysfunction in Systemic Autoimmune Diseases. Enhanced oxidative status and mitochondrial dysfunction are hallmarks of Systemic Autoimmune Diseases. These processes promote the alteration of key physiological functions such as the ability to repair vascular tissue, the control of apoptosis and the development of NETosis. The impaired functions are directly associated with tissue and organ damage in the long term. The dysregulation of the immune system leads to the loss of tolerance which drives autoantibody production, inflammation and the consequent increase of the disease activity. Furthermore, the chronic establishment of an altered oxidative status can trigger the development of cardiovascular comorbidities such as atherosclerosis and endothelial dysfunction. ROS, Reactive oxygen species.

Jointly, oxidative stress and concomitant mitochondrial dysfunction damage both, molecules and cellular structures, thus altering the correct function of organs and systems, promoting chronic tissue inflammation, dyslipidemia and atherosclerosis [48,49]. In addition, oxidative stress may promote immunomodulation, which leads autoimmune disease development [50,51] (Figure 1). Likewise, long-term treatments may promote changes in systemic oxidative stress, thereby affecting the development of these diseases.

### 2.2. NETosis as an Essential Link between Oxidative Stress and Autoimmunity

Neutrophils, key players in the immune system, are known for their antimicrobial function, being the key effectors against infections. In addition to the production of ROS and the release of microbicidal molecules, it has been described that neutrophils have an antimicrobial activity called NETosis. NETosis is a process of cell death, distinct from necrosis and apoptosis, in which, sequentially, a dissolution of inner membranes followed by chromatin decondensation occurs, along with their joint release into the extracellular space [52,53]. These webs of chromatin fibers, called neutrophil extracellular traps (NETs), contain histones, antimicrobial peptides, and oxidant-generating enzymes such as myeloperoxidase (MPO), neutrophil elastase, NOX and NO synthase (NOS).

The generation of NETs requires the production of ROS [52,54,55,56]. Thus, several mechanisms by which ROS induces NET formation have been described: (i) they lead the release of neutrophil elastase by increasing the permeability of the membrane, which in turn degrades the core histones and the linker histone H1, driving chromatin decondensation; this process is further enhanced by MPO [57]. (ii) ROS promotes the morphological changes of neutrophils occurred during NETosis [58] and (iii) ROS can induce autophagy, leading to cell membrane dissolution.

Originally, NETosis was considered just as a mechanism of immune response to infections, but numerous studies have demonstrated that these structures are key factors in many pathologic states, because they can produce tissue damage, atherosclerosis and thrombosis [59]. In addition, NETs are closely linked to autoimmunity so that several proteins in these structures, including MPO and dsDNA, may also serve as potent autoantigens [60,61]. Given the close relationship between oxidative stress and NETosis, therapies aiming at the regulation of oxidative stress also involve the reduction of NETosis, thus helping to improve the clinical status of patients with chronic and autoimmune pathologies.

### 2.3. Oxidative Stress in Antiphospholipid Syndrome

Antiphospholipid Syndrome (APS) is a systemic autoimmune disease characterized by pregnancy morbidities and/or a hypercoagulable state of the venous or the arterial vasculature, associated with the persistence of antiphospholipid antibodies (aPLs), comprising anti-beta2-glycoprotein I (anti-ß2GPI), anti-cardiolipin antibodies (aCL), and Lupus anticoagulant (LA). These patients mainly suffer vascular damage, including thrombosis, atherosclerosis, myocardial infarction or stroke [62,63].

Antiphospholipid antibodies have been demonstrated to induce an increased expression of numerous molecules involved in thrombosis development, such as prothrombotic molecules (i.e., tissue factor, the main inductor of coagulation in vivo), inflammatory mediators, cell-adhesion receptors, NETosis, and numerous intracellular signaling molecules. Besides, oxidative stress has been shown to be involved in the pathophysiology of APS, so that an enlarged oxidative status in plasma, demonstrated by high levels of prostaglandin 2 and 8-isoprostane, has been proven in APS patients, particularly those showing triple positivity for aPLs [64].

Autoantibody production has been associated with plasma oxidation, endothelial activation, and vascular disease in APS patients. aPLs provoke superoxide and NO production, which results in increased levels of plasma peroxy-nitrites [65]. In addition, titers of aCL antibodies correlated with plasma levels of F2-isoprostanes, biomarkers of in vivo lipid peroxidation [66,67]. Lower activity of para-oxonase, an antioxidant enzyme associated to high-density lipoproteins (HDL) that prevents the oxidation of low-density lipoproteins, has also been linked to the presence of arterial anomalies. In addition, HDL shows antioxidant and anti-inflammatory properties, and reduces the bioavailability of NO [68].

Antiphospholipid antibodies are also responsible for the endothelial dysfunction that characterize these patients, through the activation of transcription factors such as the nuclear factor kB and, consequently, of the induced NO synthase (iNOS). This oxidative status, in concert with the autoimmune vascular inflammation of APS patients, also promotes the formation of the oxLDL/B2GPI complexes, which further contribute to vascular injury and atherothrombosis development [69].

At molecular level, in a previous study [70] we demonstrated increased production of ROS by circulating leukocytes from APS patients (i.e., monocytes and neutrophils), associated with altered mitochondrial membrane potential, which was related to the autoimmune profile and the pro-aterothrombotic status of these patients. Moreover, in vitro studies showed that aPL-IgG promoted a redox-sensitive signaling pathway in monocytes that regulated their prothrombotic profile. Parallel studies showed that aPLs promoted the generation of superoxide in plasmacytoid dendritic cells and monocytes, which in turn upregulated the expression of TLRs 7 and 8 [71]. Overall, these data have established the key role of autoimmunity in the oxidative status present in APS patients, which leads to the development of cardiovascular disease.

More recent studies have suggested a relevant role for NETosis in the prothrombotic status of APS patients. Thus, high levels of NETs were observed in the plasma of these patients, further linked to the occurrence of thrombotic events. Moreover, it has been shown that aPLs activate neutrophils to release NETs, thus predisposing to thrombosis [72,73].

In recent years, a number of works have identified several genes involved in thrombosis, inflammation and endothelial dysfunction in APS patients (i.e., TF, PAR1, PAR2, VEGF, Flt1, TLR2, TLR4, IL8, etc.). These genes are overexpressed in immune and vascular cells (platelets, monocytes, neutrophils, and endothelial cells) and induce thrombin generation and enlarged procoagulant activity [74,75,76,77,78,79]. Microarray studies carried out by our group have also identified the altered expression in APS monocytes of several genes involved in mitochondrial biogenesis and function, antioxidant defense, and oxidative stress, all of them linked to thrombosis development [80]. Mechanistic studies further demonstrated that aPLs modulated in these cells the expression of a number of genes involved in this pro-thrombotic status, such as SLC25A27, IL-11RA, CCL2, IFIT1, PPARγ, and ARHGEF5.

More recently, the occurrence of a fine post-transcriptional regulation of gene expression has been demonstrated in autoimmune diseases. Among them, microRNAs are non-protein coding small RNAs which are ubiquitously expressed and deregulated in most pathologies, that regulate almost every cellular process [81]. Our group has characterized several microRNAs in monocytes from APS and SLE patients [82], further related to the cardiovascular feature of these pathologies. Among them, the reduced expression of miR-125a, miR-155 and miR-146a were linked to the cellular levels of peroxides, the altered mitochondrial membrane potential (ΔΨm) and the augmented activity of mitochondrial SOD. Hence, transfection of monocytes with miR-125a mimic provoked a downregulation of peroxides production, suggesting that the upregulation of these microRNAs might promote the loss of mitochondrial integrity and function, thus establishing a pro-oxidative status in APS patients.

### 2.4. Oxidative Stress in Systemic Lupus Erythematosus

Systemic lupus erythematosus (SLE) is an autoimmune disease characterized by a multisystemic inflammatory status associated with the presence of antibodies in cytoplasmic and nuclear antigens, with a relapsing and remitting course. The etiology of this disease involves genetic, immune, hormonal, and environmental dysfunctions, although molecular mechanisms explaining this altered autoimmune response are still undetermined.

Oxidative stress plays a relevant role in the pathogenesis of SLE, through the induction of inflammation, apoptosis and breaking of immune tolerance [83,84]. Thus, the interaction of macromolecules generated by apoptosis with ROS generates neo-epitopes that expand antibody flares, which contribute to tissue damage in SLE patients [85,86].

NETosis is also augmented in SLE, which, along with the reduced capacity to degrade NETs by nucleases, increases tissue damage [87]. Moreover, enzymes engaged in the NETs, such as MPO and NOX, have been shown to oxidize HDL. Hence, these lipoprotein particles acquire a pro-atherogenic form that impairs the cholesterol efflux capacity, leading to lipoprotein oxidation and increasing the cardiovascular risk in SLE [88].

The inflammatory burden of SLE, along with the elevated production of ROS and the reduced antioxidant capacity [89] may further contribute to the worsening of the disease and the occurrence of cardiovascular events. Accordingly, in biological fluids increased levels of oxidized biomolecules have been demonstrated (i.e., MDA (malondialdehyde)-modified proteins, anti-SOD and anti-catalase antibodies, and albumin modified by HNE (4- hydroxy 2-nonenal)), which correlate with disease activity and organ damage [90,91]. In addition, levels of F2-isoprostane (8-iso-PGF2), increased in urine from SLE patients, are also associated with disease activity [92]. Many independent studies have equally identified elevated levels of MDA, F2-Isoprostane, and NO, along with diminished levels of GSH in patients with lupus nephritis [93,94,95,96].

Some years ago, Lopez et al. [97] proposed that elevated levels of oxidized low-density lipoproteins (oxLDL) along with high titers of autoantibodies might act as risk factors for CVD in SLE patients. Thereafter, in a cohort of 40 SLE patients, Yailmaz et al. [98] demonstrated the occurrence of subclinical coronary microvascular dysfunction in SLE patients associated with inflammation (increased CRP levels) and impaired total antioxidant capacity of plasma.

Our group also reported altered myeloid cell activity in SLE patients, involving cell activation, inflammation and enlarged oxidative status in monocytes and neutrophils. Thus, altered expression of cell surface receptors, cytokines/chemokines, regulators of endothelial cells and molecules related to oxidative stress were found closely interrelated and associated with the development of atherosclerosis and CVD in these patients [99]. Mitochondrial dysfunction was also demonstrated in these patients, further linked to their prothrombotic and inflammatory status, as indicated by correlations with inflammatory mediators and by the association between the percentage of monocytes showing depolarized mitochondria and the occurrence of thrombotic events.

Overall, the noxious effects of ROS in SLE cause several effects: (i) shift of intracellular redox condition, i.e., decreased GSH/GSSH ratio, (ii) leukocyte mitochondrial dysfunction; (iii) oxidative modification of lipids, proteins and DNA, identified by increased levels of various biomarkers in serum and leukocytes, such as oxygen radicals (i.e., peroxides and hydroxyl radicals), oxidative-DNA damage markers (i.e., 8-oxodG), bioproducts of protein oxidation (i.e., carbonylated proteins and 3-nitrotyrosine) and bioproducts of lipid peroxidation (i.e., malondialdehyde, isoprostanes); (iv) activation of oxidative stress and gene mutations associated with antioxidant enzymes. All these alterations ultimately drive the evolution of the disease, the organ damage and the occurrence of comorbidities such as atherothrombosis.

### 2.5. Oxidative Stress in Rheumatoid Arthritis

Rheumatoid Arthritis (RA) is a systemic autoimmune disease characterized by chronic joint inflammation, which fosters cartilage and bone damage and ultimately leads to systemic involvement. Besides, RA may affect several organs outside the joints, including the lung, skin and cardiovascular system [100].

RA development involves numerous pathogenic mechanisms and clinical manifestations, even among individuals with same diagnosis and/or throughout different stages of the disease, which support its complexity and the participation of multiple factors in both the trigger and the evolution of the disease.

Oxidative stress is a key contributor to the pathogenesis of RA. ROS, overproduced in joints and circulating myeloid cells, damage cellular structures and molecules such as lipids, proteins, carbohydrates and DNA [101]. Accordingly, RA patients display increased levels of oxidation bioproducts, including lipo-peroxides (LPO), carbonylated proteins, nitro-tyrosine, and oxidized DNA [102].

Inflammation is the major pathologic mechanism of RA. Correspondingly, a close link between redox reaction producing oxidative stress and the pathophysiology of inflammation in this autoimmune disease has been demonstrated [103,104]. Thus, ROS can modify the signaling of nuclear factor κB (NF-κB), the transcription factor regulating most immune and inflammatory processes [105]. ROS can induce the translocation of NF-κB to the nucleus, while the overexpression of the antioxidant enzyme superoxide dismutase 2 (SOD2) inhibits this process [106,107]. Likewise, ROS induce other transcription factors related to cell differentiation, vascularization and proliferation, such as inducible hypoxia factor (HIF-1), activator protein 1 (AP-1) and gamma-activated peroxisome proliferator receptor (PPARγ) [108,109,110]. It has been further shown that mitochondrial ROS promote the production of key inflammatory cytokines (IL1β, IL-6, and TNFα) [111].

In turn, the inflammation process also induces oxidative stress, so that neutrophils can generate ROS by the NADPH oxidase enzyme pathway [112,113]. Therefore, through interactions among inflammation and oxidative stress, the effects of both pathogenic mechanisms are heightened.

Regarding the damage promoted by ROS in key cells involved in the pathophysiology of RA, it has been shown that ROS induce the death of chondrocytes, along with a high inflammatory injury, both at articular and systemic levels. Moreover, positive correlations between ROS and disease activity score (DAS28) have been demonstrated, supporting the hypothesis that ROS can be a predictive biomarker of the inflammatory status of RA patients [101]. Similarly, a reduced antioxidant status, also identified in these patients, is associated with a higher inflammatory damage.

Some post-translational protein modifications also occur under pro-oxidative conditions, thus generating neoepitopes identified as ‘non-self’ by the immune system that promote the formation of new autoantibodies, which, in turn, lengthen the pathologic process [114]. Likewise, we previously showed that anti-citrullinated protein antibodies (ACPAs, the most specific autoantibodies of RA) induce a pro-oxidative status in neutrophils and monocytes, in parallel to a pro-inflammatory profile in lymphocytes and monocytes [115]. Thus, ROS can be considered as biomarkers for the prediction of the inflammation and the disease activity status of RA patients.

### 2.6. Oxidative Stress in Type I Diabetes

Type 1 diabetes (T1D) is a chronic autoimmune disease mediated by T cells, which develops mainly in children and adolescents. It gradually destroys the beta cells of Langerhans islets, resulting in insufficient insulin production. The reduced circulating insulin levels result in reduced glucose uptake by the tissues, leading to intracellular hypoglycemia and extracellular hyperglycemia. Subsequently, chronic hyperglycemia can cause macrovascular and microvascular complications such as cardiovascular disease (including stroke, atherosclerosis, and myocardial infarction), neuropathy, nephropathy, and retinopathy [116].

Several factors (genetic, immunological and/or environmental) are involved in the β cell death mechanisms that lead to insulin deficiency. In addition, oxidative stress further contributes to both the worsening of the disease and inflammatory complications [117,118].

A considerable body of evidence supports the concept that T-cell mediated infiltration of the pancreas leads to generation of ROS, along with the production of proinflammatory cytokines, mainly TNFα, IL-1β and IFNγ. Thus, locally produced ROS are involved in the effector mechanisms of beta cell destruction in vitro and, correspondingly, T cells and macrophages’ secreted cytokines such as IFNγ, IL-1β and TNFα, induce the production of ROS by B-lymphocytes [119], leading to a chronic inflammatory status.

Concomitantly, the demonstrated low serum antioxidant status in T1D patients, identified by reduced urate, and vitamin C total antioxidant capacity [120,121] promote an oxidative modification of proteins and lipids [122]. Moreover, the mitochondria of β-cells have very low levels of antioxidant enzyme activity of catalase, glutathione peroxidase and superoxide dismutase [123,124] which make β-cells prone to disruption by oxidative stress and, in genetically predisposed individuals, to develop targets for a subsequent cytokine-mediated autoimmune attack.

There are also indications that oxidative stress is involved in other aspects of autoimmune diabetes including macrophage-mediated clearance of apoptotic cells and complications that develop in the vasculature of diabetic patients [125]. Likewise, vascular complications are, at least partially, dependent on hyperglycemia, which promote tissue damage by three main mechanisms: (i) increased polyol activity, which causes accumulation of fructose and sorbitol; (ii) increased generation of advanced glycation end products (AGEs); and (iii) protein kinase C activation and enlarged hexosamine pathway flux. These pathways, along with hyperglycemia induced by mitochondrial dysfunction and endoplasmic reticulum stress, induce ROS accumulation which, in turn, provokes cell damage and the worsening of the disease status. Moreover, ROS damage proteins, lipids and DNA and regulate several cellular signaling pathways, i.e., mitogen activated protein kinases and redox-sensitive transcription factors, that cause alteration in protein expression, leading to oxidative modifications that have a relevant role in vascular diseases’ development [126].

Overall, helpful therapies for T1D patients should bring not just protection from ROS of the β-cells, but also prevention of autoimmune attack mediated by ROS, which may be achieved by two main processes: (a) preventing NF-κB activation, thus reducing inflammation and the initiation of the adaptive immune response, and (b) reducing hyperglycemia that leads to disease progression and vascular involvement.

### 2.7. Oxidative Stress in Multiple Sclerosis

The central nervous system (CNS) is especially susceptible to the damage of oxidative stress due to its elevated oxygen requirement and to its high levels of lipids easily suffering peroxidation [127,128]. Neurodegenerative diseases, which are characterized by the degeneration and death of neurons that lead motor and cognitive dysfunction, are closely related to oxidative stress [129,130]. Specifically, astrocytes and microglia especially contribute to neurodegeneration [131,132]. Yet, it remains unclear whether the high levels of ROS/RNS identified in the brain of patients with neurodegenerative diseases are the cause or the consequence of that degenerative process.

Multiple Sclerosis (MS), the most frequent chronic inflammatory disease of the CNS, is characterized by completely or partially reversible episodes of worsening of the neurological functions, lasting for days or even weeks. MS is branded by a destruction of myelin, glial reaction, and chronic inflammation. It is still not known if the cause of tissue damage in MS is intrinsic or extrinsic to the CNS. In MS, several factors, such as genetic, environmental, cultural, socioeconomic, personal lifestyle, and/or aging, lead to the formation of a causative complex and ultimately converge into parallel pathognomonic clinical pictures. Nonetheless, a number of studies have demonstrated a key role for adaptive immunity in the pathogenesis of MS [133].

Inflammatory and demyelinating attacks are unique manifestations in MS, but different patho-mechanisms manage the clinical courses in each MS subtype. Among them, disturbance of redox metabolism plays a key role in the pathogenesis of MS. However, it is still unknown if the events of inflammation and neurodegeneration that characterize this disease are the cause, a complementary process, or a resultant event of ROS damage [134,135].

Proteomics analyses in serum have shown that several proteins are up-regulated in relapse-remitting MS (RRMS) patients, such as apolipoprotein E, clusterin, ceruloplasmin and complement C3. In these patients it was also demonstrated that vitamin D-binding protein had a trend to oxidation, along with increased oxidation of apolipoprotein A-IV, leading the progression from remission to relapse of MS [136].

Cerebrospinal fluid (CSF) of patients in the remission stage of RRMS also displays increased uric acid levels (a product of purine oxidation), abridged antioxidants, and augmented synthesis intrathecal of IgG [137]. Several observations have further demonstrated the presence of redox metabolism disturbance and involvement of inflammation in RRMS. Finally, lower concentrations of GSH in the frontoparietal region of patients with primary progressive MS and secondary progressive MS than in RRMS have been observed [138].

Taken together, oxidative stress in CNS is closely related to neurodegeneration in patients with progressive types of MS. Thus, the quantification of various redox components, including oxidative and antioxidative enzymes, along with the products of degradation during disease progression and degradation products during its progression, might accelerate the development of the most suitable personalized treatment plans for MS patients [139].

## 3. CoQ_10_ as a Potential Therapeutic Tool in Systemic Autoimmune Diseases

### 3.1. Antiphospholipid Syndrome and Systemic Lupus Erythematosus

**Figure 2:**
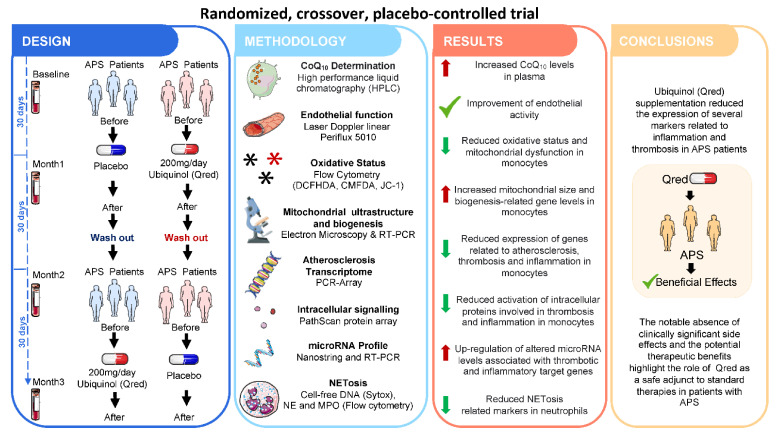
Clinical trial: Ubiquinol supplementation in antiphospholipid (APS) Patients. A randomized, crossover, placebo-controlled trial was carried out in 36 APS patients to analyze the beneficial effects of supplementation with Ubiquinol (200 mg/day) for one month. Changes in several parameters related to inflammation, oxidative stress, mitochondrial function, atherosclerosis, and NETosis, along with regulatory microRNAs, were assessed. The results demonstrated the high potential of Ubiquinol to modulate markers associated with thrombosis and cardiovascular disease in APS patients, highlighting its role as a safe adjunct to standard therapies in this autoimmune disorder.

Precedent studies by our group identified the key role of oxidative stress and mitochondrial dysfunction in plasma and leukocytes from APS patients on the establishment of their pro-atherothrombotic status. We further showed their stimulation by aPLs, and the inhibitory effects of in vitro treatment with CoQ_10_ on these processes [51,70]. Thereafter, through the implementation of a prospective, randomized, double-masked, placebo-controlled study (Clinical Trials.gov: NCT02218476), we evaluated the in vivo effects of ubiquinol supplementation to the standard therapy of APS patients on parameters related to thrombosis and inflammation. In this clinical trial, thirty-six patients were randomized to receive either, ubiquinol (200 mg/day) or placebo for 1 month [140] (Figure 2 and Table 1). 

Firstly, a direct anticoagulant effect of ubiquinol administration for one month was established, as demonstrated by the significant inhibition in APS monocytes of the expression and activity of Tissue Factor (TF), previously shown to be directly involved in the pathogenesis of thrombotic complications in APS patients. Besides, after ubiquinol treatment, plasma and cellular levels of several markers related to endothelial function, cell surface receptors, cytokines and chemokines were found jointly reversed. Likewise, a higher percentage of low-potential mitochondria in monocytes, accompanied by a lower ROS production, and the upregulation of genes related to mitochondrial biogenesis was observed in response to in vivo ubiquinol treatment. In parallel, a significant improvement of the microvascular function in patients with APS, with a significant increase in the peak flow and the area of reactive hyperemia after temporary occlusion of blood flow, was also demonstrated. Accordingly, levels of cell adhesion molecules, such as vascular cell adhesion molecule 1, were found diminished in APS patients’ plasma. A direct effect of ubiquinol in inhibiting NETosis was further demonstrated, thus suggesting a direct link between both processes. Thus, our data point at ubiquinol as a beneficial antioxidant compound that further prevents toxic side effects of NETs in inflammation and thrombosis in patients with APS.

Lastly, our results demonstrated, for the first time, an altered expression of various microRNAs by the effect of ubiquinol, most of them having as potential targets several factors involved in inflammation and thrombosis. Therefore, the increased levels of these microRNAs after ubiquinol supplementation might be associated with a reduction in the prothrombotic profile of APS patients. Accordingly, the integrated analysis of validated microRNAs and the altered genes identified by a polymerase chain reaction array of atherosclerosis demonstrated the presence of a complex network related to CVD, on which several ubiquinol-upregulated microRNAs seem to simultaneously control the expression of various ubiquinol-downregulated genes.

In summary, our results have supported the short-term impact of ubiquinol supplementation in the reduced expression of several markers related to inflammation and thrombosis in APS patients. Because of the notable absence of clinically significant side effects and its potential therapeutic benefits, this study suggested that ubiquinol might act as a safe adjunct to standard therapies in patients with APS. To the best of our knowledge, this is the only study developed up to now analyzing the beneficial effects of CoQ_10_ supplementation in the treatment of this autoimmune disorder. Nevertheless, new studies in larger cohorts are required to support their use in daily clinical practice.

In the setting of SLE, a very recent study has evaluated the potential therapeutic benefits of the CoQ_10_ analog Idebenone in two murine models of lupus [141]. In this study, MRL/lpr mice were treated orally with Idebenone at 2 different doses (1 gm/kg or 1.5 gm/kg idebenone-containing diet) for eight weeks. Activity, immunologic, clinical, and metabolic parameters were evaluated at peak disease and compared to those in untreated mice. These results were further confirmed using the lupus-prone NZM2328 mouse model. Interestingly, Idebenone-treated mice showed a significant decrease in mortality rate after eight weeks of therapy. Moreover, the treatment mitigated some disease features, such as glomerular inflammation and fibrosis, and improved renal function in association with decreased IL-17A and IL-18 renal expression. Splenic proinflammatory cytokines and inflammasome-related genes were also decreased in Idebenone treated mice, without any drug toxicity. Furthermore, Idebenone downregulated NETs formation in neutrophils and inhibited neutrophil extracellular trap formation in neutrophils from lupus-prone mice, while improving mitochondrial metabolism and ATP production, ameliorating endothelium-dependent vasorelaxation and reducing lipid peroxidation. Finally, Idebenone-treated NZM2328 mice showed a better renal function and a reduced systemic inflammation, involving reduced expression of interferon and inflammasome-related genes. In summary, the results of this study have indicated that Idebenone improves immune dysregulation, organ damage, vasculopathy, and mitochondrial function in murine SLE through pleiotropic effects on the immune compartment, immunometabolism, and tissue damage.

In a parallel study, Fortner et al. [142] investigated the therapeutic potential of the mitochondria-targeted antioxidant MitoQ on lupus disease manifestations in MRL-lpr mice. In this study, lupus-prone MRL-lpr mice were treated with MitoQ (200 µM) for eleven weeks. After therapy, reduced NETosis and ROS production were observed, along with downregulated serum levels of IFN and reduced immune complex formation in kidneys.

**Figure 3:**
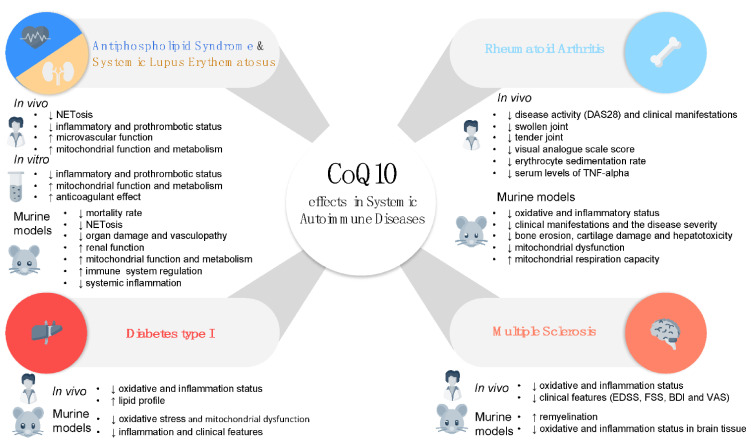
Beneficial effects of CoQ_10_ in Systemic Autoimmune Diseases. The therapeutic potential of CoQ_10_ in Systemic Autoimmune Diseases has been widely investigated. Thus, numerous studies comprising in vitro analysis, preclinical murine models and randomized controlled clinical trials have demonstrated the capacity of CoQ_10_ to improve the main clinical features of each disease, through both immunomodulatory and antioxidant effects. DAS28, Disease Activity Score using 28 joint counts; EDSS, Expanded disability status scale; FSS, Fatigue severity scale; BDI, Beck’s depression inventory; VAS, Visual analogue scale for pain.

These overall finding suggest that agents modulating mitochondrial ROS and oxidative stress, administered as adjuvant therapy to traditional immunosuppressive therapy, might have a therapeutic role in the treatment of SLE (Figure 3 and Table 1).

### 3.2. Rheumatoid Arthritis

Several preclinical studies in animal models of RA along with clinical trials in patients have demonstrated the beneficial effects of CoQ_10_ in the reduction of the oxidative and inflammatory status as well as clinical features that characterize this systemic autoimmune disease.

Jhun J. et al. showed that daily oral administration of 20 mg/kg CoQ_10_ to zymosan-induced arthritis (ZIA) for seven weeks significantly decreased the severity of RA [143]. Cartilage damage as well as the infiltration of inflammatory cells was notably lower in CoQ_10_ treated mice in relation to the vehicle. Immunohistochemical analysis of synovial tissues identified a reduction in the levels of both pro-inflammatory mediators such as TNF, IL1, IL17, IL21 and VEGF, and oxidative stress markers including iNOS and nitro-tyrosine. In addition, CoQ_10_ reduced osteoclast genesis markers such as RANK and RANK-L in joint tissues which was in line with the decrease of bone erosion and cartilage damage noticed in the mouse model of RA. CoQ_10_ also exhibited immunomodulatory effects on B and T-cells. Markers of germinal center B cells such as CD220, CD138 and GL-7 were reduced along with the levels of total IgG, IgG1 and IgG2a in CoQ_10_ treated mice. Moreover, markers of T follicular helper cells were also decreased including PD-1, ICOS and GL-7. In spleen tissues, CoQ_10_ promoted the downregulation of Th17-cells, the inhibition of pSTAT-3 in these cells, and the up-regulation of Foxp3-expressing Treg cells.

The immunomodulatory effects of CoQ_10_ have also been shown in other mouse models of RA like collagen-induced arthritis (CIA). In a very recent study, the oral administration of CoQ_10_ encoded in liposome/gold hybrid nanoparticles (LGNP CoQ_10_) for 10 weeks showed the reduction of the pathology scores of RA including bone damage, cartilage damage and immune cells infiltration [144]. The levels of pro-inflammatory cytokines such as IL6, TNF, IL17 and IL1 were reduced in the joints of CoQ_10_-treated mice. Similar effects related to the reduction of Th17 and P-STAT-3 cells as well as osteoclast genesis markers were also identified in this animal model.

In another study using CIA mice, Lee et al. demonstrated that the combination of CoQ_10_ with other dietary supplements such as Zinc and a probiotic complex reduced the disease severity through inhibition of immune response [145]. Total IgG, IgG1 and IgG2a levels were reduced in the serum of mice treated with CoQ_10_. Besides, the downregulation of pro-inflammatory cytokines such as IL17, IL6, and IFN, and the up-regulation of the anti-inflammatory cytokine IL10 were observed after CoQ_10_ treatment. In line with previous studies, CoQ_10_ also affected the reciprocal balance of Th17/Treg, increasing Treg differentiation and decreasing Th17 differentiation.

The synergic effect of CoQ_10_ and drugs with anti-inflammatory and antioxidant properties has also been demonstrated in preclinical models of RA. Jhun et al. treated CIA mice with metformin (Met) (1 mg/mouse), CoQ_10_ (0.1 mg/mouse), the combination of both of them, and anti-TNF therapy [146]. The combination of Met and CoQ_10_ resulted in a lower degree of cartilage damage and inflammation than Met, CoQ_10_ and anti-TNF therapy alone. The combination of Met and CoQ_10_ also promoted the reduction of pro-inflammatory cytokines such as TNF, IL6, and IL1 in the joints compared with the other treatments alone. Besides, the drug combination significantly reduced the levels of total IgG and the proportion of Th-17 cells and induced the differentiation of Foxp3 T-reg cells. Regarding the altered oxidative status presented in the CIA model, the combination of Met and CoQ_10_ suppressed mitochondrial dysfunction, reduced the expression of nitro-tyrosine and increased mitochondrial respiration capacity. These results were also more prominent when Met and CoQ_10_ were used in combination compared with Met, CoQ_10_ and anti-TNF alone.

Bauerova et al. demonstrated that the combination of CoQ_10_ with methotrexate (MTX), the most commonly prescribed disease-modifying anti-rheumatic drugs, suppressed the progression of RA in rats more effectively than MTX alone [147]. Adjuvant arthritis (AA) was induced by the injection of Mycobacterium butyricum in rats. CoQ_10_ was supplemented in a daily oral dose of 20 mg/kg, while MTX was administered orally at a dose of 0.3 mg/kg twice a week. AA rats with MTX, CoQ_10_ or the combination of MTX and CoQ_10_ were evaluated on days 14, 21 and 28. The combination therapy was the most effective in reducing the hind paw volume of arthritic animals on all selected days. These results were in line with the reduction of oxidative stress markers in plasma close to the control group such as protein carbonyls and MDA- and HNE-protein adducts by the combination of MTX and CoQ_10_ therapy. Regarding immunomodulatory effects of these therapies, the functionality of peripheral blood neutrophils including phagocytosis, oxidative burst and metabolic activity was improved with the combination therapy, which also reduced the levels of IL1 in plasma. All the effects were more pronounced with the combination of MTX and CoQ_10_ than MTX or CoQ_10_ alone.

Tawfik et al. validated the anti-arthritic effect of the combination of MTX and CoQ_10_ in the AA model and further explored the protective role of CoQ_10_ in the hepatotoxicity induced by MTX [148]. Liver markers such as the serum levels of Alanine Aminotransferase (SGPT), Aspartate Aminotransferase (SGOT), Alkaline phosphatase (ALP), and total bilirubin were improved after the combined treatment of MTX with CoQ_10_ compared with MTX alone. Furthermore, oxidative stress markers and inflammatory cytokines altered by the MTX therapy in the liver were ameliorated when MTX was combined with CoQ_10_. These results were confirmed by the histopathological evaluation of the liver where the combination therapy attenuated the intensity of liver injury such as necrosis and inflammation promoted by MTX therapy in the AA model. These results highlighted the dual role of CoQ_10_ in improving the efficacy and minimizing the adverse effects of MTX.

The potential of CoQ_10_ as useful therapeutic tool in RA exhibited by preclinical studies in several mouse models of RA has also been confirmed in a randomized, double blind, placebo-controlled trial in patients with RA. Abdollahzad et al. conducted a clinical trial in 44 patients with moderate activity of RA who received either 100 mg/day of CoQ_10_ (n = 22) or placebo (n = 22) for two months [149]. Blood samples were collected before and after the intervention to evaluate changes in oxidative and inflammatory markers. Adverse effects were not observed during the intervention. The supplementation with CoQ_10_ promoted a reduction in the serum levels of malondialdehyde (MDA) which was not observed in the placebo group. However total antioxidant capacity in serum did not change between both groups of patients. Furthermore, the serum levels of TNF-alpha were significantly reduced in RA patients supplemented with CoQ_10_ compared with the placebo group, while a trend towards a reduction in IL6 levels was also noticed.

In line with these results, Nachvak et al. have recently conducted another clinical trial in 54 RA patients where the effect of CoQ_10_ in clinical and serological features of RA has been evaluated [150]. RA patients with high activity were randomly assigned into two groups of 27 subjects to receive either 100 mg/day of CoQ_10_ or placebo for two months. The clinical assessment of these patients after the intervention revealed that the supplementation with CoQ_10_ significantly reduced the disease activity score (DAS28) (from 5 to 2.34), the swollen joint count (from 2 to 0), the tender joint count (from 4.5 to 0) and the visual analogue scale (VAS) score. Complementary analysis including the erythrocyte sedimentation rate and the serum level of matrix metalloproteinase-3 (MMP3) was also significantly reduced after the intervention. These changes were not observed in the placebo group.

Altogether, these results support the beneficial effect of CoQ_10_ in the control of the oxidative and inflammatory status of RA patients which is translated in the amelioration of the disease activity and their clinical manifestations (Figure 3 and Table 1). Large additional clinical trials in independent cohorts are needed to validate these results, as well as to evaluate its potential synergic effects with disease-modifying anti-rheumatic drugs. These approaches might favor the establishment of CoQ_10_ as a complementary therapeutic tool for the future management of RA.

### 3.3. Type I Diabetes

The capacity of CoQ_10_ to reduce oxidative stress and inflammation and ameliorate other clinical features associated with T1D like neuropathic pain was revealed in a pre-clinical mouse model of T1D induced by streptozotocin [151]. As neural tissue is especially vulnerable to oxidative stress associated with chronic hyperglycemia, samples from dorsal root ganglia, sciatic nerves, and spinal cord, as well as serum, were obtained after the intervention with CoQ_10_ (150 mg/kg). Hyperglycemia induced an increase in lipid peroxide levels. However, mice treated with CoQ_10_ exhibited a reduction of lipid peroxide levels in serum, dorsal root ganglia and sciatic nerves which reflect its antioxidant capacity. Regarding the analysis of the inflammatory status, hyperglycemic mice showed an increase in the levels of inflammatory mediators such as p65 (the activated marker of nuclear factor-κB) and CCL2. Mice treated with CoQ_10_ presented a reduction in the levels of these inflammatory mediators in dorsal root ganglia. The anti-inflammatory properties of CoQ_10_ in this tissue were also confirmed at protein and mRNA levels. These results were in line with the prophylactic and antinociceptive effects of CoQ_10_ on the diabetic neuropathic pain developed by this T1D mouse model. Accordingly, in an experimental model of type 2 diabetic nephropathy (db/db), which showed a deficiency in mitochondrial oxidized CoQ_10_, its supplementation preserved renal mitochondrial dysfunction by reducing both excess renal mitochondrial hydrogen peroxide production and mitochondrial membrane potential [152].

In patients with T1D, several studies have also evaluated the potential of CoQ_10_ supplementation to reduce the oxidative and inflammatory status present in this disease. Montano et al. conducted a study in which 13 T1D patients were supplemented with 100 mg of Ubiquinone (CoQ_10_) twice daily for 12 weeks [153]. The levels of CoQ_10_ were significantly increased in serum of these patients after the supplementation and were directly associated with an improvement in the redox balance and lipid profile. Thus, the plasma concentrations of oxidized LDL and total cholesterol were significantly reduced after CoQ_10_ supplementation, while serum levels of redox regulating protein glutaredoxin 1 (Grx1) activity and total antioxidant capacity were increased, leading to a less oxidant extracellular environment.

Brauner et al. supplemented 23 T1D and T2D patients with 100 mg of CoQ_10_ twice daily for 12 weeks and evaluated changes in antimicrobial peptides and NK cells, both components of the innate immune response associated with diabetes [154]. After the supplementation, the levels of human beta-defensin-2 (hBD2) were strongly reduced in serum of T1D patients. Furthermore, CoQ_10_ induced changes in subset distribution and activation markers of NK cells, improving their activity in T1D patients.

These results highlight the potential of CoQ_10_ supplementation in the control of key features of T1D, such as the altered immune response and redox status associated with the disease (Figure 3 and Table 1). However, clinical trials aiming to evaluate the role of CoQ_10_ in the control of blood glucose in patients with T1D have failed so far [155,156].

### 3.4. Multiple Sclerosis

Supplementation with CoQ_10_ in a pre-clinical mouse model of multiple sclerosis (MS) has recently demonstrated its capacity to enhance remyelination and regulate inflammation. Khalilian et al. studied the demyelination-remyelination process in a well-stablished model of MS induced by Cuprizone (CPZ) intoxication [157]. MS mice were treated orally with 150 mg/kg of CoQ_10_ for eight weeks in combination with CPZ which started four weeks earlier. Control groups included MS mice treated with CPZ for 12 weeks and healthy mice. Histological analysis of some brain regions like corpus callosum (CC) evidenced the reduction of myelin levels in CPZ mice. CoQ_10_ supplementation reversed these effects, attenuating the destructive damage exhibited by CPZ. Moreover, genes related to remyelination such as oligodendrocyte transcription factor-1 (Olig1) and myelin basic protein (MBP) were significantly increased in the brain tissue of the CoQ_10_ group. These results were supported by changes in behavior of these mice, as determined by tall suspension and open field test. Besides, CoQ_10_ promoted changes in the oxidative and inflammatory status altered in the MS model. Thus, CoQ_10_ promoted the increase of the superoxide dismutase (SOD) activity and total antioxidant capacity, and the reduction of pro-inflammatory cytokines such as IL6 and TNF in mice brain tissue.

Recently, Moccia et al. performed an open-label crossover design in 60 relapsing-remitting patients with MS to evaluate the effect of CoQ_10_ supplementation [158]. Thirty patients were treated with interferon beta1a alone and 30 patients were treated with interferon in combination with 200 mg/day of CoQ_10_ for three months. The oxidative status was markedly improved after CoQ_10_ supplementation including an increase in markers of free radical scavenging activity (uric acid and bilirubin) and a reduction of intracellular ROS and DNA damage (8-OHdG). The inflammatory profile was also modulated by CoQ_10_ in the serum of patients with MS. The levels of anti-inflammatory mediators such as IL4 and IL13 were increased, while a panel of pro-inflammatory molecules including TNF-alpha, IFN-gamma, IL-1-alpha, IL-2R, IL17F, IL-9, MIP-1α, eotaxin, RANTES, HGF and VEGF was reduced after three months of CoQ_10_ supplementation. Along with these molecular effects, CoQ_10_ also promoted changes in clinical features of MS evidenced by the significant reduction of Expanded Disability Status Scale (EDSS), Fatigue severity scale (FSS), Beck’s depression inventory (BDI) II and Visual Analogue Scale (VAS) for pain.

Sanoobar et al. conducted a randomized, double-blinded, placebo-controlled trial in 50 patients with MS to evaluate the effect of CoQ_10_. Several results were obtained and published in three different works where the beneficial effect of CoQ_10_ in the oxidative status [159], the pro-inflammatory profile [56] and clinical features [57] were highlighted. Patients were supplemented with 500 mg/day of CoQ_10_ (n = 24) or placebo (n = 24) for 12 weeks. CoQ_10_ supplementation promoted a significant increase in antioxidant markers such as SOD activity and total antioxidant capacity, as well as a reduction of oxidative markers such as malondialdehyde (MDA) levels in the serum of patients with MS [55]. Furthermore, the analysis of pro-inflammatory mediators in the serum of these patients revealed that CoQ_10_ ameliorated the inflammatory status of MS patients through the reduction of the levels of TNFα, IL-6 and MMP9 compared with the placebo group [160]. Finally, clinical features of patients with MS such as fatigue or depression were also improved after the intervention noticed by the reduction of FSS and BDI respectively [161].

Overall, CoQ_10_ supplementation has demonstrated the potential to improve clinical and molecular features of patients with MS (Figure 3). However larger and long-term follow-up studies are needed to validate and reinforce its protective role in this disease.

## 4. Conclusions

Oxidative stress damages both molecules and cellular structures, thus altering the correct function of organs and systems, inducing tissue inflammation, dyslipidemia and atherosclerosis. In addition, oxidative stress may foster immunomodulation, which leads to the development of chronic autoimmune diseases. Likewise, long-term treatments may promote changes in systemic oxidative stress. A role for mitochondrial dysfunction has been further proposed in the immune dysregulation and organ damage characteristic of autoimmune diseases.

In the search for potential therapeutic tools that modify mitochondrial function and immunometabolism in these disorders, including antiphospholipid syndrome, systemic lupus erythematosus, rheumatoid arthritis, diabetes mellitus or multiple sclerosis, experimental models and clinical trials have examined the safety and efficacy of CoQ_10_, a vital component of the mitochondrial respiratory chain with a crucial role in ATP production that acts as an antioxidant with cell-protective effects, and has been demonstrated to be helpful in numerous chronic and cardiovascular diseases.

These studies have shown that CoQ_10_ supplementation to standard therapy ameliorates the main symptoms that characterize autoimmunity, namely inflammation, vascular endothelial dysfunction, and cardiovascular risk. Moreover, after CoQ_10_ therapy, along with a global decline of disease activity, each autoimmune pathology benefits from specific improvements to damaged organs (i.e., kidneys in SLE patients, cartilage, joints and synovial tissue in RA), or even to progress in neurological involvement (i.e., in depression in multiple sclerosis).

Hence, CoQ_10_ can be considered as an important coadjuvant in the treatment of chronic conditions affecting the immune system. Nevertheless, few randomized trials analyzing the effects of CoQ_10_ or its derivatives in reducing disease progression and improving quality of life have been developed to date in the setting of autoimmunity. Thus, more focused trials on larger cohorts of autoimmune disorders are needed in order to authenticate the promising effects of CoQ_10_.

## Figures and Tables

**Table 1:** Mitochondrial evaluation of the response to Coenzyme Q_10_ and derivatives in Systemic autoinmune disorders.

COQ_10_-Related Compounds	Systemic Autoimmune Disease	Mitochondrial Process Evaluated	Laboratory Test Used	References
Ubiquinol	Antiphospholipid Syndrome (Clinical trial)	Mitochondrial membrane potential (ΔΨm) Enzymatic activity Biogenesis Fision/Fusion and regulatory proteins	Flow cytometry Enzymatic activity assays Electron microscopy Confocal fluorescence microscopy RT-PCR/Western blot (WB)	Perez-Sanchez et al., Blood. 2012;119(24):5859-70 Perez-Sanchez et al., Arterioscler Thromb Vasc Biol 2017. 2017;37(10):1923–1932
Idebenone	Systemic Lupus Erythematosus (MRL/lpr mice model)	Mitochondrial metabolism and adenosine triphosphate (ATP) production Synthesis of ROS	Mitochondrial metabolism analysis by Seahorse Fluorescence microscopy RT-PCR	Blanco LP, et al., Arthritis Rheumatol. 2020 Mar;72(3):454–464
Mito Q	Systemic Lupus Erythematosus (MRL/lpr mice model)	Mitochondrial metabolism Antiviral stimulator (MAVS) Protein oligomerisation Mitochondrial morphology	Mitochondrial metabolism analysis by Seahorse Western blot Electron microscopy	Fortner KA, et al., Lupus science & medicine 2020; 7, (1):e000387
CoQ_10_ + Metformin	Rheumatoid Arthritis (CIA mice model)	Mitochondrial membrane potential (ΔΨm) Mitochondrial metabolism	Fluorescence microscopy Mitochondrial metabolism analysis by Seahorse	Jhun J, et al. Immunopharmacol Immunotoxicol. 2016;38(2):103–112
CoQ_10_	Diabetes (db/db mouse model)	Mitochondrial membrane potential (ΔΨm)/ROS production Enzymatic activities (SOD, GPx, citrate synthase)	Flow cytometry Enzymatic activity assays	Sourris KC, et al., Free Radic Biol Med. 2012 Feb 1;52(3):716–723
